# Serum Folate in Relation to Lipid Abnormalities in Community-Dwelling Adults: A Population-Based Cross-Sectional Study in Zhejiang Province, China

**DOI:** 10.3390/nu18122024

**Published:** 2026-06-22

**Authors:** Xiangyu Chen, Jingjing Lin, Lijin Chen, Weiyuan Yao, Jieming Zhong, Mingbin Liang

**Affiliations:** Department of Non-Communicable Disease Control and Prevention, Zhejiang Provincial Center for Disease Control and Prevention, Hangzhou 310051, China; xychen@cdc.zj.cn (X.C.); jjlin@cdc.zj.cn (J.L.); ljch@cdc.zj.cn (L.C.); ywyao@cdc.zj.cn (W.Y.); mbliang@cdc.zj.cn (M.L.)

**Keywords:** serum folate, lipids, hypertriglyceridemia, low high-density lipoprotein cholesterol, cross-sectional study, China

## Abstract

**Objectives**: This study aimed to examine the cross-sectional associations between serum folate concentrations and four lipid abnormality subtypes among community-dwelling adults in Zhejiang Province, China. **Methods**: This population-based cross-sectional study included 3254 adults from Zhejiang Province, China. Serum folate concentrations were analyzed both as quartiles and per 1-standard deviation (SD) increments. Multivariable logistic regression models were used to evaluate the associations of serum folate with hypercholesterolemia, hypertriglyceridemia, high low-density lipoprotein cholesterol (LDL-C), and low high-density lipoprotein cholesterol (HDL-C). Restricted cubic spline (RCS) regression models were further applied to assess dose–response patterns. Additional RCS analyses using continuous lipid parameters were also performed. False discovery rate (FDR) correction, exploratory subgroup analyses, and sensitivity analyses were additionally conducted. **Results**: The prevalences of hypercholesterolemia, hypertriglyceridemia, high LDL-C, and low HDL-C were 7.87%, 17.12%, 4.30%, and 4.46%, respectively. In the fully adjusted model, each 1-SD increment in serum folate was associated with lower odds of hypertriglyceridemia (OR = 0.82, 95% CI: 0.73–0.92) and low HDL-C (OR = 0.63, 95% CI: 0.49–0.81). Compared with the lowest quartile, participants in the highest serum folate quartile had lower odds of hypertriglyceridemia (OR = 0.62, 95% CI: 0.46–0.82) and low HDL-C (OR = 0.38, 95% CI: 0.22–0.64), with significant trends across quartiles (both *p* for trend < 0.001). No significant associations were observed for hypercholesterolemia or high LDL-C. These findings remained significant after FDR correction. RCS analyses suggested an overall inverse association between serum folate and hypertriglyceridemia, with no evidence of nonlinearity (*p* for overall = 0.001; *p* for nonlinearity = 0.212), whereas the association with low HDL-C showed evidence of nonlinearity (*p* for overall < 0.001; *p* for nonlinearity = 0.009). Additional RCS analyses using continuous lipid parameters showed broadly consistent findings for TG and HDL-C. Exploratory subgroup and sensitivity analyses showed generally similar results. **Conclusions**: Higher serum folate concentrations were cross-sectionally associated with lower odds of hypertriglyceridemia and low HDL-C among community-dwelling adults in Zhejiang Province, China, whereas no significant associations were observed for hypercholesterolemia or high LDL-C. Further prospective cohort studies are warranted to verify these cross-sectional findings and to explore underlying mechanisms.

## 1. Introduction

Cardiovascular disease (CVD) remains the leading cause of death worldwide, and dyslipidemia is a major modifiable risk factor contributing to the development of atherosclerotic cardiovascular disease [1]. Abnormal lipid metabolism, characterized by elevated total cholesterol (TC), triglycerides (TG), and low-density lipoprotein cholesterol (LDL-C) as well as reduced high-density lipoprotein cholesterol (HDL-C), has been consistently associated with increased risks of coronary heart disease, stroke, and other metabolic disorders [2,3,4]. In China, rapid urbanization, dietary westernization, and increasingly sedentary lifestyles have contributed to a substantial rise in the prevalence of dyslipidemia during recent decades, posing an important public health challenge [5]. Identifying nutritional factors associated with lipid abnormalities may help improve understanding of lipid-related cardiometabolic risk.

Folate, an essential water-soluble B vitamin, functions as a key cofactor in one-carbon metabolism and methylation reactions [6]. Although its role in homocysteine metabolism has been extensively studied, accumulating evidence suggests that folate may also participate in lipid metabolism through several biological pathways [7,8]. Previous studies have shown that folate deficiency may impair phosphatidylcholine synthesis through the phosphatidylethanolamine *N*-methyltransferase pathway [9,10], which is required for very-low-density lipoprotein (VLDL) assembly and hepatic lipid export [11]. Disturbance of this pathway may subsequently influence circulating TG and cholesterol concentrations [12,13]. In addition, folate may reduce oxidative stress and lipid peroxidation, preserve lipoprotein receptor activity, and modulate inflammatory responses involved in lipid metabolism [14,15]. These mechanisms may partly explain the potential association between folate status and lipid abnormalities.

Despite these potential mechanisms, epidemiological and interventional studies have yielded inconsistent findings regarding the relationship between folate and lipid metabolism. Several observational studies have reported that higher folate concentrations are associated with lower TG levels and higher HDL-C levels, suggesting a potentially favorable lipid profile [16,17]. Some randomized controlled trials and meta-analyses have also demonstrated modest reductions in TG and TC following folic acid supplementation [18,19,20]. However, other studies have reported null associations [21]. In particular, recent evidence from East Asian populations has suggested possible non-linear associations between folate status and lipid parameters, indicating that both low and high folate concentrations may be associated with unfavorable lipid profiles [22]. These inconsistent findings may partly be explained by differences in population characteristics, dietary patterns, folic acid fortification policies, folate exposure levels, and adjustment for important confounding factors across studies.

China provides a unique setting for investigating the association between folate and lipid abnormalities because large-scale mandatory folic acid fortification of staple foods has not been implemented, unlike in several Western countries [23]. Consequently, serum folate concentrations in the Chinese population primarily reflect natural dietary intake and individual nutritional status. Compared with dietary folate intake estimated from questionnaires, serum folate concentrations may more directly reflect individual folate status and bioavailability. This characteristic allows evaluation across a wider range of naturally occurring folate exposure levels. Nevertheless, several important gaps remain in the current literature. First, population-based evidence regarding serum folate and specific lipid abnormality subtypes among Chinese adults remains limited. Second, although several studies have reported associations between folate and lipid metabolism, evidence regarding potential dose–response relationships between serum folate and different lipid abnormality subtypes remains insufficient, particularly for non-linear associations.

Zhejiang Province, one of the most economically developed regions in Eastern China, has experienced a rapidly increasing burden of metabolic disorders accompanying lifestyle transition and population aging. However, evidence regarding the association between serum folate concentrations and lipid abnormalities among community-dwelling adults in this region remains scarce. Therefore, the present study aimed to examine the associations between serum folate concentrations and specific lipid abnormality subtypes among community-dwelling adults in Zhejiang Province, China, using data from a population-based cross-sectional study.

## 2. Materials and Methods

### 2.1. Study Design and Participants

**Figure 1:**
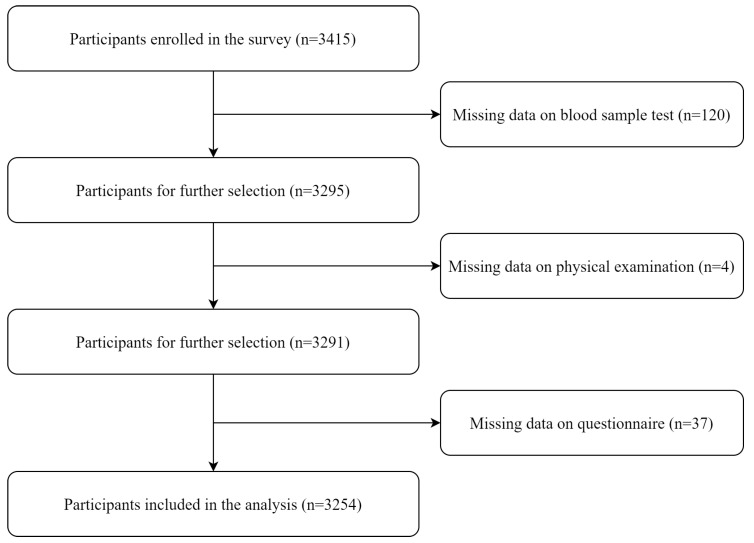
Flowchart of the participant selection process.

This cross-sectional survey was conducted between March and May 2019 in five townships (Heping Town, Lijiaxiang Town, Meishan Town, Lincheng Town, and Taihu Town) of Changxing County, Huzhou City, Zhejiang Province, China. Permanent residents aged 30 years or older who had lived in the local area for at least six months prior to the survey were eligible for inclusion. Individuals with incapacity for civil conduct and pregnant women were excluded. All participants underwent face-to-face interviews using a structured questionnaire administered via Android-based tablets, physical examinations (including measurements of height and weight), blood pressure assessment, and fasting venous blood sampling for laboratory measurements. The participant selection process is illustrated in Figure 1. Participants with missing blood sample, physical examination, or questionnaire data were excluded from the analysis.

### 2.2. Data Collection

Trained staff from local primary healthcare institutions conducted face-to-face questionnaire interviews using Android tablets. They also performed standardized anthropometric measurements, including height and body weight, as well as blood pressure (BP) measurements. BP was measured twice using a validated electronic sphygmomanometer (OMRON J7136, Omron Healthcare Co., Ltd., Kyoto, Japan) after a 5 min rest, and the average of the two readings was used for analysis.

Venous blood samples were collected from all participants in the morning after an overnight fast of at least 12 h, according to a standardized protocol. Participants were instructed to refrain from food and caloric beverages during the fasting period. Individuals who did not meet the fasting requirement were excluded from laboratory testing. No self-reported deviations from the fasting protocol were recorded during data collection. Laboratory assays included serum folate, serum vitamin B12, fasting plasma glucose (FPG), total cholesterol (TC), triglycerides (TG), low-density lipoprotein cholesterol (LDL-C), and high-density lipoprotein cholesterol (HDL-C). Serum folate and serum vitamin B12 were measured using liquid chromatography–tandem mass spectrometry. For serum folate, the quality-control coefficients of variation were 9.56% at the low concentration level and 8.10% at the high concentration level. The limit of detection was 1.13 nmol/L. Instrument hardware calibration was performed annually, and standard curve calibration was conducted for each analytical batch using matrix-matched serum calibrators with at least six non-zero concentration points covering clinically relevant decision levels. Daily instrument performance was checked using system suitability testing with three consecutive injections. Internal quality control was performed after every 40 samples, and external quality assessment was conducted through regular participation in external quality assessment programs. Serum lipids were analyzed using enzymatic methods, and FPG was determined by the hexokinase method. All biochemical analyses were conducted by a certified testing laboratory following standardized laboratory protocols and quality-control procedures.

### 2.3. Definition of Variables

According to the 2016 Chinese guidelines for the management of dyslipidemia [24], hypercholesterolemia was defined as TC ≥ 6.2 mmol/L, hypertriglyceridemia as TG ≥ 2.3 mmol/L, high LDL-C as LDL-C ≥ 4.1 mmol/L, and low HDL-C as HDL-C < 1.0 mmol/L. Age was categorized into younger than 60 years and 60 years or older. Educational level was dichotomized into primary school or lower and middle school or higher. Body mass index (BMI) was calculated as weight in kilograms divided by height in meters squared (kg/m^2^) and analyzed both continuously and categorically according to Chinese criteria (normal weight: <24 kg/m^2^; overweight/obesity: ≥24 kg/m^2^) [25]. Hypertension was defined as systolic blood pressure (SBP) ≥ 140 mmHg and/or diastolic blood pressure (DBP) ≥ 90 mmHg, or a self-reported physician-diagnosed hypertension [26]. Diabetes was defined as FPG ≥ 7.0 mmol/L, or self-reported physician-diagnosed diabetes [27]. Smoking status was defined as current smoker and non-current smoker; drinking status as regular alcohol consumption and non-regular drinking during the past year; and regular exercise was defined as engaging in moderate to vigorous physical activity for at least 30 min per session, five or more days per week (i.e., at least 150 min per week).

### 2.4. Statistical Methods

Continuous variables were presented as mean ± standard deviation (SD) or median (interquartile range [IQR]), and categorical variables were presented as frequencies and percentages. All analyses were conducted using complete-case data. Participants with missing laboratory, physical examination, or questionnaire data were excluded before statistical analyses, and no imputation was performed because the missing information was completely unavailable for these participants. Baseline characteristics were compared using Student’s *t* test or the Mann–Whitney U test for continuous variables, as appropriate, and the chi-square test for categorical variables. Multicollinearity among covariates was assessed using variance inflation factors (VIFs), and all VIF values were below 2, indicating no substantial multicollinearity. Multivariable logistic regression models were used to examine the associations between serum folate and four lipid abnormality subtypes, including hypercholesterolemia, hypertriglyceridemia, high LDL-C, and low HDL-C. Serum folate was analyzed both as a continuous variable (per 1-SD increment) and as quartiles (Q1–Q4), with the lowest quartile (Q1) serving as the reference group. The quartile cut-off values were 12.08, 18.76, and 28.60 nmol/L. Three sequential models were constructed: Model 1 was unadjusted; Model 2 was adjusted for age and sex; and Model 3 was additionally adjusted for education level, BMI, serum vitamin B12, hypertension, diabetes, smoking, alcohol drinking, regular exercise, and lipid-lowering therapy. Trends across quartiles were assessed by modeling the median value within each quartile as a continuous variable. To account for multiple testing across the four primary lipid abnormality outcomes, Benjamini–Hochberg false discovery rate (FDR) correction was applied to the *p*-values for serum folate per 1-SD increment in the fully adjusted model. Restricted cubic spline (RCS) regression models with four knots were used to examine potential nonlinear associations between serum folate and each lipid abnormality subtype. The knots were placed at the 5th, 35th, 65th, and 95th percentiles of the serum folate distribution. The models were adjusted for the same covariates included in Model 3, and the median serum folate concentration was used as the reference value. In addition, RCS analyses using linear regression models were performed with continuous TC, TG, LDL-C, and HDL-C concentrations as outcomes to further evaluate the associations between serum folate and lipid parameters. Exploratory subgroup analyses were conducted to assess the consistency of the associations between serum folate (per 1-SD increment) and lipid outcomes that showed significant associations in the primary analyses, namely hypertriglyceridemia and low HDL-C. Predefined subgroups included age (<60, ≥60 years), sex (male, female), BMI (<24, ≥24 kg/m^2^), hypertension (yes, no), diabetes (yes, no), smoking status (yes, no), and drinking status (yes, no). For each subgroup analysis, the fully adjusted model was applied after excluding the corresponding stratification variable. Potential interaction effects were evaluated by including multiplicative interaction terms between serum folate and each stratification variable in the fully adjusted models. Several sensitivity analyses were performed to assess the robustness of the primary findings. First, the multivariable logistic regression and RCS analyses were repeated after excluding participants receiving lipid-lowering therapy. Second, the fully adjusted model was repeated after removing serum vitamin B12 from the adjustment set. Third, the fully adjusted model was repeated without adjustment for lipid-lowering therapy. Fourth, to further address potential residual confounding by available dietary and socioeconomic factors while avoiding excessive model complexity, an extended adjustment model was constructed by additionally including vegetable intake, staple food intake, and self-rated economic status in addition to the covariates included in Model 3. Vegetable intake and staple food intake were self-reported and recorded in liang, a traditional Chinese unit of weight equivalent to 50 g, and were therefore included as crude dietary proxy variables rather than precise measures of dietary folate intake or total energy intake. Finally, to assess the stability of estimates for outcomes with relatively low event counts, Firth penalized logistic regression was performed for high LDL-C and low HDL-C using the same covariates as those included in Model 3. All statistical tests were two-sided, and a *p*-value < 0.05 was considered statistically significant. All analyses were performed using R software (version 4.2.2; R Foundation for Statistical Computing, Vienna, Austria).

## 3. Results

### 3.1. General Characteristics of the Participants

A total of 3254 adults were included in the study. The mean age was 61.91 ± 6.50 years, and 58.64% of participants were female. The prevalence of hypercholesterolemia was 7.87%, hypertriglyceridemia was 17.12%, high LDL-C was 4.30%, and low HDL-C was 4.46%. Most participants had a low educational level, with 74.40% having completed primary school education or below, and 52.64% were classified as overweight or obese. The prevalence rates of hypertension and diabetes were 66.20% and 16.29%, respectively. Regarding lifestyle factors, 22.25% of participants were current smokers, 31.81% reported regular alcohol consumption, and 4.49% engaged in regular physical exercise. The median serum folate concentration was 18.76 nmol/L, while the median serum vitamin B12 concentration was 154.98 pmol/L. Detailed participant characteristics are presented in Table 1.

### 3.2. Basic Characteristics of the Subjects According to Lipid Abnormality Status

Table 2 presents participant characteristics according to four lipid abnormality subtypes: hypercholesterolemia, hypertriglyceridemia, high LDL-C, and low HDL-C. Compared with their corresponding reference groups, participants with hypertriglyceridemia were younger, had higher BMI, and had higher prevalences of hypertension and diabetes (all *p* < 0.001). Participants with high LDL-C and low HDL-C also had significantly higher BMI (both *p* < 0.01). Hypertension was more prevalent among those with high LDL-C (*p* < 0.01), whereas diabetes was more common among those with low HDL-C (*p* < 0.05). Serum folate concentrations were significantly lower in participants with hypertriglyceridemia and low HDL-C (both *p* < 0.001), whereas no significant differences were observed for hypercholesterolemia or high LDL-C. Serum vitamin B12 concentrations were lower only in the hypertriglyceridemia group (*p* < 0.001). Sex differences were observed for hypercholesterolemia and low HDL-C, while regular exercise did not differ significantly across lipid abnormality subtypes. Detailed participant characteristics are presented in Table 2.

### 3.3. Multivariable Logistic Regression Analysis of Serum Folate in Relation to Four Lipid Abnormality Subtypes

Table 3 summarizes the associations between serum folate levels and the odds of four lipid abnormality subtypes, including hypercholesterolemia, hypertriglyceridemia, high LDL-C, and low HDL-C, using multivariable logistic regression models. For hypertriglyceridemia, each 1-SD increment in serum folate was associated with an 18% lower odds in the fully adjusted model (OR = 0.82, 95% CI: 0.73–0.92). A graded inverse association was observed across quartiles. Compared with Q1, the adjusted ORs were 0.86 (95% CI: 0.67–1.12) for Q2, 0.74 (95% CI: 0.56–0.97) for Q3, and 0.62 (95% CI: 0.46–0.82) for Q4, with a significant trend across quartiles (*p* for trend < 0.001). For low HDL-C, an inverse association was also observed. Each 1-SD increase in serum folate was associated with a 37% lower odds (OR = 0.63, 95% CI: 0.49–0.81). Compared with Q1, the fully adjusted ORs for Q2, Q3, and Q4 were 0.52 (95% CI: 0.34–0.81), 0.44 (95% CI: 0.27–0.71), and 0.38 (95% CI: 0.22–0.64), respectively, with a significant trend across quartiles (*p* for trend < 0.001). For hypercholesterolemia and high LDL-C, no significant associations with serum folate were observed. In the fully adjusted model, the ORs per 1-SD increment in serum folate were 0.94 (95% CI: 0.82–1.08, *p* = 0.390) for hypercholesterolemia and 1.03 (95% CI: 0.86–1.22, *p* = 0.777) for high LDL-C. Likewise, no significant associations were observed across quartiles or in the trend analyses (all *p* for trend > 0.05).

To account for multiple testing across the four primary lipid abnormality outcomes, Benjamini–Hochberg false discovery rate (FDR) correction was applied to the *p*-values for serum folate per 1-SD increment in the fully adjusted model. The findings remained consistent with the primary analysis: the associations with hypertriglyceridemia and low HDL-C remained statistically significant after FDR correction, whereas the associations with hypercholesterolemia and high LDL-C remained non-significant (Table S1).

### 3.4. Dose–Response Analysis of Serum Folate in Relation to Different Lipid Abnormality Subtypes

**Figure 2:**
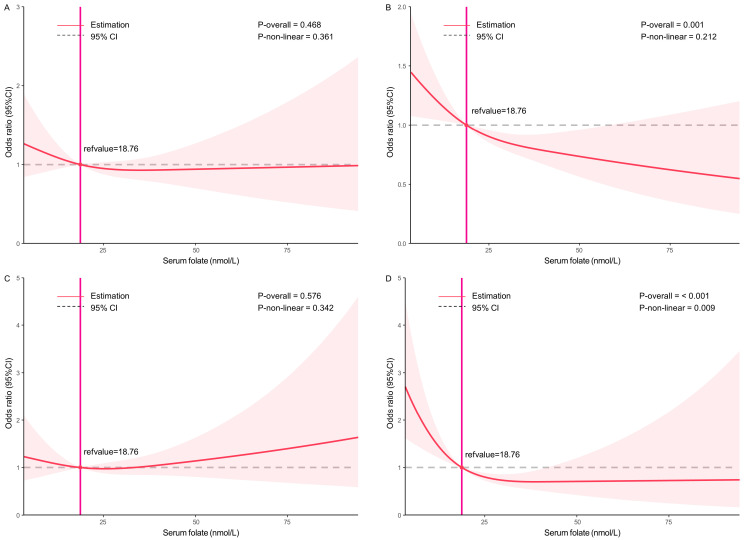
Dose–response relationships between serum folate and lipid outcomes assessed using restricted cubic spline (RCS) models. RCS regression models with four knots were used to examine the dose–response relationships between serum folate and lipid abnormality subtypes, including hypercholesterolemia, hypertriglyceridemia, high low-density lipoprotein cholesterol (LDL-C), and low high-density lipoprotein cholesterol (HDL-C), with adjustment for age, sex, education level, body mass index (BMI), smoking, drinking, hypertension, diabetes, serum vitamin B12, regular exercise, and lipid-lowering therapy. Odds ratios (ORs) and 95% confidence intervals (CIs) were estimated from logistic regression models. Serum folate levels (nmol/L) are shown on the *x*-axis, and ORs for each lipid outcome are shown on the *y*-axis. The solid red lines represent the fitted spline curves, and the shaded areas indicate the 95% confidence intervals. The horizontal dashed line indicates an OR of 1 (no association). Vertical solid lines indicate the 50th percentile (median; reference value) of the serum folate distribution. (**A**) Association between serum folate and hypercholesterolemia. (**B**) Association between serum folate and hypertriglyceridemia. (**C**) Association between serum folate and high LDL-C. (**D**) Association between serum folate and low HDL-C. Abbreviations: OR, odds ratio; CI, confidence interval.

To further evaluate the shape of the associations observed in the multivariable logistic regression analyses, RCS regression models with four knots were constructed after adjustment for age, sex, education level, BMI, smoking, drinking, hypertension, diabetes, serum vitamin B12, regular exercise, and lipid-lowering therapy. As shown in Figure 2, serum folate exhibited distinct dose–response patterns across lipid abnormality subtypes. For hypertriglyceridemia (Figure 2B), an overall inverse association was observed between serum folate and hypertriglyceridemia, with the odds of hypertriglyceridemia gradually decreasing as serum folate levels increased and no evidence of nonlinearity (*p* for overall = 0.001; *p* for nonlinearity = 0.212). For low HDL-C (Figure 2D), the association was statistically significant overall and showed evidence of nonlinearity (*p* for overall < 0.001; *p* for nonlinearity = 0.009). The odds decreased more rapidly at lower serum folate concentrations and then tended to plateau at higher concentrations. Consistent with the multivariable logistic regression results, no significant overall associations were observed between serum folate and hypercholesterolemia (Figure 2A) or high LDL-C (Figure 2C) (both *p* for overall > 0.05).

To make fuller use of continuous lipid measurements, additional RCS analyses based on linear regression models were performed using TC, TG, LDL-C, and HDL-C concentrations as continuous outcomes. As shown in Figure S1, serum folate showed significant nonlinear associations with TG and HDL-C (both *p* for nonlinearity < 0.05), whereas no significant overall associations were observed for TC or LDL-C (both *p* for overall > 0.05). The curves suggested lower TG levels and higher HDL-C levels mainly within the lower range of serum folate concentrations, followed by relatively flatter patterns at higher concentrations. Although the shape of the association differed between binary hypertriglyceridemia and continuous TG analyses, the overall direction of the findings was consistent with the primary analyses based on lipid abnormality subtypes.

### 3.5. Results of Exploratory Subgroup Analyses

**Figure 3:**
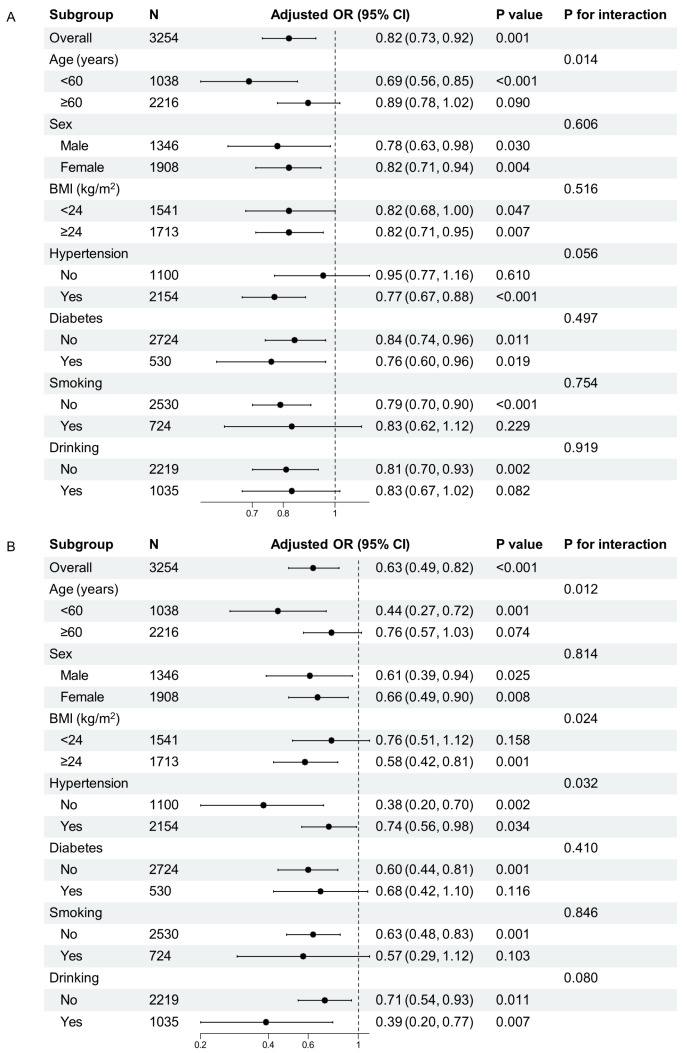
Subgroup analyses for the association between serum folate and lipid outcomes (per SD increment). Subgroups included age, sex, body mass index (BMI), hypertension, diabetes, smoking, and drinking status. Odds ratios and 95% confidence intervals were estimated from fully adjusted models, with the corresponding stratification variable excluded from each model. Models were adjusted for age, sex, education level, BMI, smoking, drinking, hypertension, diabetes, regular exercise, serum vitamin B12, and lipid-lowering therapy. The black dots represent OR point estimates, horizontal lines indicate 95% confidence intervals, and the vertical dashed line indicates the null value (OR = 1). (**A**) Subgroup analysis for hypertriglyceridemia. (**B**) Subgroup analysis for low HDL-C. Abbreviations: SD, standard deviation; BMI, body mass index; OR, odds ratio; CI, confidence interval.

Given the significant associations observed for hypertriglyceridemia and low HDL-C in the primary analyses, exploratory subgroup analyses were performed to evaluate the consistency of these associations across different population subgroups. Interactions were assessed by including multiplicative interaction terms in the fully adjusted models, with adjustment for age, sex, education level, BMI, serum vitamin B12, hypertension, diabetes, smoking, alcohol drinking, regular exercise, and lipid-lowering therapy. As shown in Figure 3A, the inverse association between serum folate and hypertriglyceridemia was generally consistent across most subgroups. An interaction signal was observed for age (*p* for interaction = 0.014), with a stronger association among participants aged < 60 years than among those aged ≥ 60 years. Significant inverse associations were identified in both males and females, participants with BMI ≥ 24 kg/m^2^, individuals with hypertension, non-smokers, non-drinkers, and participants with or without diabetes (all *p* < 0.05). For low HDL-C (Figure 3B), interaction signals were observed for age, BMI, and hypertension (all *p* for interaction < 0.05). The association between serum folate and low HDL-C appeared stronger among younger participants, non-hypertensive individuals, and those with BMI ≥ 24 kg/m^2^. Significant associations were also detected in both sexes, participants with hypertension, non-smokers, drinkers, non-drinkers, and non-diabetic individuals (all *p* < 0.05). Because multiple subgroup comparisons were conducted and some strata had relatively small sample sizes, these subgroup findings should be considered exploratory and interpreted with caution. Detailed subgroup results are presented in Figure 3A,B.

### 3.6. Sensitivity Analyses

Several sensitivity analyses were conducted to assess the robustness of the primary findings. First, after excluding participants receiving lipid-lowering therapy (*n* = 3181), the associations between serum folate and lipid abnormality subtypes remained largely consistent with the primary analyses (Table S2). In the fully adjusted model, each 1-SD increment in serum folate remained significantly associated with lower odds of hypertriglyceridemia (OR = 0.79, 95% CI: 0.70–0.89) and low HDL-C (OR = 0.61, 95% CI: 0.47–0.80). No significant associations were observed for hypercholesterolemia or high LDL-C. The RCS analyses after excluding lipid-lowering therapy users showed similar patterns, with an inverse linear association for hypertriglyceridemia (*p* for nonlinearity > 0.05) and a nonlinear inverse association for low HDL-C (*p* for nonlinearity < 0.05), whereas no significant overall associations were observed for hypercholesterolemia or high LDL-C (both *p* for overall > 0.05) (Figure S2).

Second, the results were materially unchanged when vitamin B12 was removed from the adjustment model (Table S3). Serum folate per 1-SD increment remained significantly associated with hypertriglyceridemia (OR = 0.80, 95% CI: 0.71–0.90) and low HDL-C (OR = 0.63, 95% CI: 0.49–0.81), while no significant associations were observed for hypercholesterolemia or high LDL-C. Similarly, when lipid-lowering therapy was not included as an adjustment variable, the results remained consistent with the primary analyses (Table S4). Serum folate per 1-SD increment was still significantly associated with hypertriglyceridemia (OR = 0.82, 95% CI: 0.73–0.92) and low HDL-C (OR = 0.63, 95% CI: 0.49–0.82).

Third, to further address potential residual confounding by available dietary and socioeconomic factors, an extended adjustment model was additionally constructed by further including vegetable intake, staple food intake, and self-rated economic status in addition to the covariates included in Model 3. The results remained generally consistent with the primary analyses (Table S5). In this extended model, serum folate per 1-SD increment remained significantly associated with lower odds of hypertriglyceridemia (OR = 0.81, 95% CI: 0.72–0.91) and low HDL-C (OR = 0.65, 95% CI: 0.51–0.84). Compared with the lowest serum folate quartile, participants in the highest quartile also had lower odds of hypertriglyceridemia (OR = 0.60, 95% CI: 0.45–0.80) and low HDL-C (OR = 0.39, 95% CI: 0.23–0.66), with significant trends across quartiles (both *p* for trend < 0.001). No significant associations were observed for hypercholesterolemia or high LDL-C.

Finally, to assess the stability of estimates for outcomes with relatively low event counts, Firth penalized logistic regression analyses were performed for high LDL-C and low HDL-C. The results were similar to those from the primary logistic regression analyses: no significant association was observed for high LDL-C, whereas the association between serum folate per 1-SD increment and low HDL-C remained statistically significant (Table S6). Overall, these sensitivity analyses supported the robustness of the main findings.

## 4. Discussion

In this population-based cross-sectional study of community-dwelling adults in Zhejiang Province, China, higher serum folate concentrations were cross-sectionally associated with lower odds of hypertriglyceridemia and low HDL-C after adjustment for demographic characteristics, lifestyle factors, serum vitamin B12, and lipid-lowering therapy. No significant associations were observed for hypercholesterolemia or high LDL-C. These findings remained statistically significant for hypertriglyceridemia and low HDL-C after FDR correction. Dose–response analyses further showed an inverse linear association between serum folate and hypertriglyceridemia, whereas the association with low HDL-C appeared nonlinear, with a steeper decrease in odds at lower serum folate concentrations followed by a flatter pattern at higher concentrations. Additional RCS analyses using continuous lipid parameters showed broadly consistent results, with significant associations observed for TG and HDL-C but not for TC or LDL-C. Sensitivity analyses, including analyses excluding lipid-lowering therapy users, analyses without adjustment for vitamin B12 or lipid-lowering therapy, an extended adjustment model additionally including available dietary and socioeconomic variables, and Firth penalized logistic regression for outcomes with low event counts, also supported the stability of the main findings.

The present findings are generally consistent with several previous epidemiological studies reporting associations between folate status and lipid profiles, particularly for TG and HDL-C. For instance, in a Chinese hypertensive population, participants with low serum folate levels had a higher likelihood of hypertriglyceridemia and low HDL-C than those with normal folate status after adjustment for multiple covariates [28]. Similar findings were reported in a U.S. study using National Health and Nutrition Examination Survey (NHANES) data, in which higher serum folate concentrations were associated with lower TG and higher HDL-C levels after excluding folic acid supplement users [6]. A German retrospective analysis also reported a positive relationship between plasma folate and HDL-C [16]. However, findings for TC and LDL-C have been less consistent. A Korean nationally representative study reported a U-shaped association between serum folate and both hypercholesterolemia and elevated LDL-C, while the prevalence of low HDL-C decreased as serum folate increased [22]. These differences may be related to differences in folate distributions, folic acid fortification policies, supplement use, dietary patterns, genetic background, and baseline metabolic risk across study populations.

In the present study, serum folate was mainly associated with hypertriglyceridemia and low HDL-C, whereas no significant associations were observed for hypercholesterolemia or high LDL-C. This subtype-specific pattern may suggest that folate status is more closely related to lipid abnormalities linked to triglyceride metabolism and HDL-C metabolism than to cholesterol-related outcomes. Several biological pathways may be relevant to these observed associations, although they remain speculative because intermediate biomarkers were not measured. Folate is involved in one-carbon metabolism and methyl donor availability, which are related to phosphatidylcholine synthesis through the phosphatidylethanolamine *N*-methyltransferase pathway [29]. Phosphatidylcholine is needed for hepatic very-low-density lipoprotein (VLDL) assembly and secretion [30]. Experimental and mechanistic evidence suggests that insufficient folate status may be related to impaired hepatic triglyceride export, intracellular lipid accumulation, and altered circulating TG metabolism [31]. In addition, HDL-C metabolism is closely linked to triglyceride-rich lipoprotein turnover and hepatic lipid handling [32]. Therefore, disturbances in hepatic triglyceride transport and VLDL metabolism may also be related to lower HDL-C concentrations [33]. By comparison, TC and LDL-C are more strongly regulated by cholesterol synthesis, intestinal cholesterol absorption, and LDL receptor activity [34]. These pathways may be less directly linked to folate-related one-carbon metabolism, which may be one possible reason for the absence of significant associations for hypercholesterolemia and high LDL-C in this study.

The dose–response analyses provided further information on the shape of these associations. For hypertriglyceridemia, the association with serum folate appeared approximately linear, suggesting lower odds of hypertriglyceridemia across higher serum folate concentrations within the observed range. For low HDL-C, the association appeared nonlinear, with a sharper decrease in odds at lower folate concentrations and a flatter pattern at higher concentrations. This pattern may suggest that the observed association between serum folate and HDL-C-related abnormality is stronger in the lower folate range. However, this finding should be interpreted cautiously because confidence intervals widened at higher folate concentrations, suggesting lower precision in the upper tail of the folate distribution. Additional RCS analyses using continuous lipid parameters showed significant nonlinear associations for TG and HDL-C, while no significant overall associations were observed for TC or LDL-C. Although the curve shape differed between binary hypertriglyceridemia and continuous TG analyses, the direction of the findings was consistent, showing that serum folate was mainly related to TG-related and HDL-C-related outcomes.

The exploratory subgroup analyses suggested broadly consistent inverse associations of serum folate with hypertriglyceridemia and low HDL-C across several population subgroups. Some interaction signals were observed, including age for hypertriglyceridemia and age, BMI, and hypertension for low HDL-C. Several explanations may account for these findings. Younger individuals may have fewer cumulative metabolic disturbances and lower disease burden, allowing the relationship between folate and lipid metabolism to be more readily detected [35]. In overweight or obese individuals, insulin resistance, hepatic lipid accumulation, and increased VLDL production are common metabolic abnormalities [36]. Under these conditions, one-carbon metabolism and phosphatidylcholine synthesis may become increasingly important for maintaining normal hepatic lipid transport and triglyceride handling. Consequently, variation in folate status may have greater metabolic relevance among individuals with excess adiposity [37]. However, these subgroup findings should be interpreted with caution because multiple subgroup comparisons were conducted, and some strata had relatively small sample sizes. Therefore, these findings should be viewed as exploratory rather than confirmatory. Future studies with larger sample sizes and more balanced subgroup distributions are needed to assess whether age, adiposity, or hypertension status modifies the association between folate status and lipid abnormalities.

The sensitivity analyses supported the stability of the main findings. After excluding participants receiving lipid-lowering therapy, the associations of serum folate with hypertriglyceridemia and low HDL-C remained statistically significant, while no significant associations were observed for hypercholesterolemia or high LDL-C. Similar results were observed when vitamin B12 was removed from the adjustment model and when lipid-lowering therapy was not included as a covariate. In the extended adjustment model, further including available dietary and socioeconomic variables, including vegetable intake, staple food intake, and self-rated economic status, the main findings also remained generally consistent. These analyses reduce concern that the observed findings were mainly driven by model specification, adjustment for vitamin B12 or lipid-lowering therapy, or incomplete adjustment for available dietary and socioeconomic factors. In addition, Firth penalized logistic regression analyses for high LDL-C and low HDL-C yielded results similar to the primary logistic regression models, suggesting that the findings were not materially affected by sparse-event bias. Nevertheless, the low event counts for high LDL-C and low HDL-C still require cautious interpretation, especially for the null association with high LDL-C.

The nutritional context of the Chinese population may also be relevant when interpreting these findings. Unlike several Western countries that have implemented mandatory folic acid fortification [38], China has not adopted nationwide mandatory folic acid fortification policies [23]. Therefore, serum folate concentrations in this study may mainly reflect natural dietary intake and individual nutritional status. This setting may allow assessment across a broader naturally occurring range of serum folate concentrations. In populations with mandatory folic acid fortification, serum folate distributions may shift toward higher levels with less variation, which could affect the observed associations between folate status and lipid outcomes.

From a public health perspective, our findings suggest cross-sectional associations between serum folate status and lipid abnormalities, particularly hypertriglyceridemia and low HDL-C, which are common lipid features related to cardiometabolic risk in Asian populations [39]. However, because of the cross-sectional design, these findings should not be interpreted as evidence that higher folate status prevents lipid abnormalities. The present study also cannot support conclusions regarding folic acid supplementation or dietary intervention. Prospective studies and intervention studies with detailed dietary, supplement, and biomarker data are needed before any public health recommendations can be made.

This study has several strengths. First, it was based on a relatively large community-based population from Eastern China. Second, serum folate concentrations were directly measured, which may better reflect circulating folate status than dietary assessment alone. Third, multiple statistical approaches were used, including multivariable logistic regression, RCS analysis, FDR correction, exploratory subgroup analysis, and several sensitivity analyses. Fourth, important covariates, including serum vitamin B12 and lipid-lowering therapy, were considered in the analyses.

Several limitations should also be acknowledged. First, because of the cross-sectional study design, temporal relationships and causality cannot be established. Reverse causation remains possible, as participants with dyslipidemia may have changed their dietary behaviors or supplement use after diagnosis, which could affect serum folate concentrations. Therefore, the direction of the observed associations cannot be determined. Second, although multiple potential confounders were adjusted for, residual confounding cannot be fully excluded. Vegetable intake and staple food intake were available, but they were self-reported and recorded in liang (1 liang = 50 g), which provided only crude estimates of dietary intake. Therefore, these variables could not precisely capture dietary folate intake, total energy intake, macronutrient composition, or overall dietary patterns. Self-rated economic status was also available, but it may not fully represent socioeconomic status. Although folic acid supplement use was collected, only 2 participants reported use; therefore, it was not included as a separate covariate to avoid sparse-data problems. Other potentially important factors, including liver function, renal function, thyroid function, inflammatory markers, and homocysteine concentrations, were not measured in the present study. The lack of homocysteine data also limited our ability to explore possible biological mechanisms. In addition, information on medications such as antihypertensive agents, antidiabetic drugs, including metformin, and anticonvulsants was unavailable, which may also have introduced residual confounding because these medications may affect folate metabolism or lipid profiles. Regular exercise was assessed by self-report, and the low prevalence of regular exercise may partly reflect underreporting or misclassification, particularly because light, household, occupational, or informal physical activity may not have been captured by the predefined criterion. Therefore, residual confounding related to physical activity cannot be fully excluded. Third, serum folate mainly reflects relatively short-term folate status and may not fully represent long-term folate exposure. Fourth, the prevalence of high LDL-C and low HDL-C was relatively low, resulting in limited outcome events and reduced statistical precision. Although Firth penalized logistic regression yielded results similar to the primary analyses, modest associations, particularly for high LDL-C, may have gone undetected. In addition, treatment-related outcome misclassification cannot be completely excluded because some participants receiving lipid-lowering therapy may have had lipid concentrations below diagnostic thresholds at the time of blood testing. Finally, the study was conducted in a single county in Zhejiang Province, and the study population was mainly older adults with a relatively low educational level. Therefore, selection bias and limited generalizability cannot be excluded. The findings may not be directly generalizable to younger, urban, or more highly educated populations, or to populations in countries with mandatory folic acid fortification.

## 5. Conclusions

In conclusion, higher serum folate concentrations were cross-sectionally associated with lower odds of hypertriglyceridemia and low HDL-C among community-dwelling adults in Zhejiang Province, China, whereas no significant associations were observed for hypercholesterolemia or high LDL-C. These findings remained generally consistent after FDR correction, additional RCS analyses using continuous lipid parameters, exploratory subgroup analyses, and several sensitivity analyses. The results suggest differential cross-sectional associations between serum folate and lipid abnormality subtypes, with stronger associations observed for TG-related and HDL-C-related outcomes than for cholesterol-related outcomes. Given the cross-sectional design, causal inference cannot be made. Future prospective cohort studies and mechanistic studies with detailed dietary, supplement, medication, and biomarker data are needed to verify these findings and clarify the temporal and biological links between folate status and lipid metabolism.

## Figures and Tables

**Table 1:** Basic characteristics of the participants (*n* = 3254).

Characteristics	Overall (*n* = 3254)
Age (years), [means ± SD]	61.91 ± 6.50
Age group (years), *n* (%)	
<60	1038 (31.90)
≥60	2216 (68.10)
Sex, *n* (%)	
Male	1346 (41.36)
Female	1908 (58.64)
Educational level, *n* (%)	
Primary school or lower	2421 (74.40)
Middle school or higher	833 (25.60)
BMI (kg/m^2^), [means ± SD]	24.38 ± 3.41
BMI category (kg/m^2^), *n* (%)	
<24	1541 (47.36)
≥24	1713 (52.64)
SBP (mmHg), [means ± SD]	141.17 ± 19.22
DBP (mmHg), [means ± SD]	82.97 ± 10.96
Hypertension, *n* (%)	2154 (66.20)
FPG (mmol/L), [means ± SD]	5.86 ± 1.48
Diabetes, *n* (%)	530 (16.29)
TC (mmol/L), [means ± SD]	4.95 ± 0.96
TG (mmol/L), [median (IQR)]	1.33 (0.93, 1.91)
HDL-C (mmol/L), [means ± SD]	1.63 ± 0.44
LDL-C (mmol/L), [means ± SD]	2.83 ± 0.72
Hypercholesterolemia, *n* (%)	256 (7.87)
Hypertriglyceridemia, *n* (%)	557 (17.12)
Low HDL-C, *n* (%)	145 (4.46)
High LDL-C, *n* (%)	140 (4.30)
Lipid-lowering therapy, *n* (%)	73 (2.24)
Smoking, *n* (%)	724 (22.25)
Drinking, *n* (%)	1035 (31.81)
Regular exercise, *n* (%)	146 (4.49)
Serum folate (nmol/L), [median (IQR)]	18.76 (12.10, 28.64)
Serum vitamin B12 (pmol/L), [median (IQR)]	154.98 (110.70, 236.16)

Abbreviations: SD, standard deviation; IQR, interquartile range; BMI, body mass index; SBP, systolic blood pressure; DBP, diastolic blood pressure; FPG, fasting plasma glucose; TC, total cholesterol; TG, triglycerides; LDL-C, low-density lipoprotein cholesterol; HDL-C, high-density lipoprotein cholesterol.

**Table 2:** Characteristics of participants according to lipid abnormality status (*n* = 3254).

Characteristics	Hypercholesterolemia			Hypertriglyceridemia			High LDL-C			Low HDL-C		
	No (*n* = 2998)	Yes (*n* = 256)	*p*	No (*n* = 2697)	Yes (*n* = 557)	*p*	No (*n* = 3114)	Yes (*n* = 140)	*p*	No (*n* = 3109)	Yes (*n* = 145)	*p*
Age (years), [means ± SD]	61.86 ± 6.51	62.50 ± 6.34	0.127	62.13 ± 6.42	60.86 ± 6.75	<0.001 ***	61.90 ± 6.49	62.19 ± 6.66	0.598	62.00 ± 6.43	60.05 ± 7.64	0.003 **
Sex, *n* (%)			0.009 **			0.734			0.056			0.038 *
Male	1260 (42.03)	86 (33.59)		1112 (41.23)	234 (42.01)		1299 (41.71)	47 (33.57)		1274 (40.98)	72 (49.66)	
Female	1738 (57.97)	170 (66.41)		1585 (58.77)	323 (57.99)		1815 (58.29)	93 (66.43)		1835 (59.02)	73 (50.34)	
BMI (kg/m^2^), [means ± SD]	24.35 ± 3.44	24.68 ±3.07	0.141	24.12 ± 3.43	25.63 ±3.05	<0.001 ***	24.34 ± 3.43	25.17 ±2.97	0.005 **	24.30 ± 3.41	25.97 ± 3.00	<0.001 ***
Hypertension, *n* (%)	1977 (65.94)	177 (69.14)	0.299	1721 (63.81)	433 (77.74)	<0.001 ***	2045 (65.67)	109 (77.86)	0.003 **	2049 (65.91)	105 (72.41)	0.105
Diabetes, *n* (%)	486 (16.21)	44 (17.19)	0.685	390 (14.46)	140 (25.13)	<0.001 ***	506 (16.25)	24 (17.14)	0.779	497 (15.99)	33 (22.76)	0.031 *
Smoking, *n* (%)	682 (22.75)	42 (16.41)	0.019 **	593 (21.99)	131 (23.52)	0.429	699 (22.45)	25 (17.86)	0.201	688 (22.13)	36 (24.83)	0.445
Drinking, *n* (%)	951 (31.72)	84 (32.81)	0.719	847 (31.41)	188 (33.75)	0.279	998 (32.05)	37 (26.43)	0.162	999 (32.13)	36 (24.83)	0.065
Regular exercise, *n* (%)	131 (4.37)	15 (5.86)	0.269	123 (4.56)	23 (4.13)	0.654	142 (4.56)	4 (2.86)	0.341	140 (4.50)	6 (4.14)	0.836
Serum folate (nmol/L), [median (IQR)]	18.74 (12.15, 28.66)	18.97 (11.42, 28.40)	0.927	19.22 (12.40, 29.16)	16.86 (10.94, 25.49)	<0.001 ***	18.74 (12.10, 28.64)	19.41 (12.15, 28.34)	0.883	18.99 (12.33, 28.85)	13.91 (9.13, 22.05)	<0.001 ***
Serum vitamin B12 (pmol/L), [median (IQR)]	154.98 (110.70, 236.16)	162.36 (118.08, 223.25)	0.304	154.98 (110.70, 243.54)	140.22 (103.32, 191.88)	<0.001 ***	154.98 (110.70, 236.16)	154.98 (103.32, 223.25)	0.767	154.98 (110.70, 236.16)	147.60 (103.32, 206.64)	0.397

* *p* < 0.05; ** *p* < 0.01; *** *p* < 0.001. Abbreviations: BMI, body mass index; SD, standard deviation; IQR, interquartile range; LDL-C, low-density lipoprotein cholesterol; HDL-C, high-density lipoprotein cholesterol.

**Table 3:** Multivariable regression analysis of serum folate in relation to four lipid abnormality subtypes (*n* = 3254).

Characteristics	Model 1		Model 2		Model 3	
	OR (95% CI)	*p*	OR (95% CI)	*p*	OR (95% CI)	*p*
Hypercholesterolemia						
Serum folate per SD increment	1.01 (0.89–1.14)	0.898	0.95 (0.83–1.09)	0.449	0.94 (0.82–1.08)	0.390
Serum folate quartile						
Q1 (*n* = 810)	1.00 (Reference)		1.00 (Reference)		1.00 (Reference)	
Q2 (*n* = 815)	0.79 (0.55–1.14)	0.214	0.73 (0.50–1.06)	0.095	0.72 (0.49–1.04)	0.082
Q3 (*n* = 814)	1.01 (0.71–1.43)	0.953	0.88 (0.61–1.25)	0.469	0.86 (0.60–1.23)	0.410
Q4 (*n* = 815)	0.87 (0.61–1.24)	0.443	0.72 (0.49–1.05)	0.084	0.69 (0.47–1.02)	0.061
*p* for trend		0.718		0.194		0.150
Hypertriglyceridemia						
Serum folate per SD increment	0.80 (0.72–0.89)	<0.001 ***	0.80 (0.72–0.90)	<0.001 ***	0.82 (0.73–0.92)	<0.001 ***
Serum folate quartile						
Q1 (*n* = 810)	1.00 (Reference)		1.00 (Reference)		1.00 (Reference)	
Q2 (*n* = 815)	0.89 (0.70–1.14)	0.348	0.87 (0.68–1.12)	0.277	0.86 (0.67–1.12)	0.258
Q3 (*n* = 814)	0.74 (0.58–0.96)	0.022 *	0.73 (0.56–0.95)	0.019 *	0.74 (0.56–0.97)	0.027 *
Q4 (*n* = 815)	0.62 (0.47–0.80)	<0.001 ***	0.61 (0.46–0.80)	<0.001 ***	0.62 (0.46–0.82)	0.001 **
*p* for trend		<0.001 ***		<0.001 ***		<0.001 ***
High LDL-C						
Serum folate per SD increment	1.06 (0.90–1.25)	0.475	1.01 (0.85–1.20)	0.900	1.03 (0.86–1.22)	0.777
Serum folate quartile						
Q1 (*n* = 810)	1.00 (Reference)		1.00 (Reference)		1.00 (Reference)	
Q2 (*n* = 815)	0.96 (0.59–1.57)	0.880	0.89 (0.55–1.47)	0.660	0.87 (0.53–1.43)	0.590
Q3 (*n* = 814)	1.15 (0.72–1.84)	0.564	1.02 (0.63–1.65)	0.950	1.01 (0.62–1.65)	0.958
Q4 (*n* = 815)	0.99 (0.61–1.61)	0.979	0.84 (0.51–1.40)	0.512	0.86 (0.51–1.44)	0.562
*p* for trend		0.938		0.575		0.666
Low HDL-C						
Serum folate per SD increment	0.62 (0.48–0.77)	<0.001 ***	0.65 (0.50–0.82)	<0.001 ***	0.63 (0.49–0.81)	<0.001 ***
Serum folate quartile						
Q1 (*n* = 810)	1.00 (Reference)		1.00 (Reference)		1.00 (Reference)	
Q2 (*n* = 815)	0.53 (0.35–0.82)	0.004 **	0.54 (0.35–0.84)	0.006 **	0.52 (0.34–0.81)	0.004 **
Q3 (*n* = 814)	0.42 (0.26–0.67)	<0.001 ***	0.44 (0.27–0.71)	<0.001 ***	0.44 (0.27–0.71)	<0.001 ***
Q4 (*n* = 815)	0.36 (0.22–0.58)	<0.001 ***	0.39 (0.23–0.65)	<0.001 ***	0.38 (0.22–0.64)	<0.001 ***
*p* for trend		<0.001 ***		<0.001 ***		<0.001 ***

* *p* < 0.05; ** *p* < 0.01; *** *p* < 0.001. Abbreviations: SD, standard deviation; LDL-C, low density lipoprotein cholesterol; HDL-C, high density lipoprotein cholesterol; OR, odds ratio; CI, confidence interval; Q1, first quartile; Q2, second quartile; Q3, third quartile; Q4, fourth quartile. Model 1: unadjusted analysis (no covariates included); Model 2: adjusted for age, sex; Model 3: fully adjusted model, incorporating age, sex, education level, body mass index, serum vitamin B12, hypertension, diabetes, smoking, alcohol drinking, regular exercise, and lipid-lowering therapy.

## Data Availability

The data presented in this study are available on request from the corresponding author (the data are not publicly available due to privacy restrictions).

## Supplementary Materials

The following supporting information can be downloaded at: https://www.mdpi.com/article/10.3390/nu18122024/s1, Table S1: False discovery rate-adjusted *p*-values for the associations between serum folate (per 1-SD increase) and lipid abnormality subtypes (*n* = 3254); Table S2: Sensitivity analysis of the associations between serum folate and lipid abnormality subtypes after excluding participants using lipid-lowering therapy (*n* = 3181); Table S3: Sensitivity analysis of the associations between serum folate and lipid abnormality subtypes without adjustment for vitamin B12 (*n* = 3254); Table S4: Sensitivity analysis of the associations between serum folate and lipid abnormality subtypes without adjustment for lipid-lowering therapy (*n* = 3254); Table S5: Sensitivity analysis of the associations between serum folate and lipid abnormality subtypes using an extended adjustment model (*n* = 3254); Table S6: Original and Firth-corrected logistic regression results for the associations of serum folate per 1-SD increment with high LDL-C and low HDL-C (*n* = 3254); Figure S1: Dose–response relationships between serum folate and continuous lipid parameters; Figure S2: Dose–response relationships between serum folate and four lipid abnormality subtypes after excluding lipid-lowering therapy users.

## Abbreviations

The following abbreviations are used in this manuscript:

TC	Total cholesterol
TG	Triglycerides
LDL-C	Low-density lipoprotein cholesterol
HDL-C	High-density lipoprotein cholesterol
VLDL	Very-low-density lipoprotein
CVD	Cardiovascular disease
RCS	Restricted cubic spline
BMI	Body mass index
BP	Blood pressure
SBP	Systolic blood pressure
DBP	Diastolic blood pressure
FPG	Fasting plasma glucose
VIF	Variance inflation factor
FDR	False discovery rate
SD	Standard deviation
IQR	Interquartile range
OR	Odds ratio
CI	Confidence interval
Q1	First quartile
Q2	Second quartile
Q3	Third quartile
Q4	Fourth quartile
NHANES	National Health and Nutrition Examination Survey
