# Supplementary material for: Serum Folate in Relation to Lipid Abnormalities in Community-Dwelling Adults: A Population-Based Cross-Sectional Study in Zhejiang Province, China
Source: Nutrients. 2026 Jun 22;18(12):2024. doi: 10.3390/nu18122024 (PMC13304543; doi:10.3390/nu18122024)
Supplement: Supplementary file 1 [file nutrients-18-02024-s001.zip › nutrients-4372630-supplementary.pdf]

Supplementary:

Table S1. False discovery rate–adjusted *p* values for the associations between serum folate (per 1-SD increase) and lipid abnormality subtypes (*n*=3,254)

Outcome	Original *p* value	FDR-adjusted *p* value
Hypercholesterolemia	0.390	0.520
Hypertriglyceridemia	<0.001***	0.001**
High LDL-C	0.777	0.777
Low HDL-C	<0.001***	0.001**

** $p<0.01$; *** $p<0.001$.

Abbreviations: SD, standard deviation; LDL-C, low density lipoprotein cholesterol; HDL-C, high density lipoprotein cholesterol; FDR, false discovery rate.

Adjusted for age, sex, education level, body mass index, serum vitamin B12, hypertension, diabetes, smoking, alcohol drinking, regular exercise and lipid-lowering therapy.

Table S2. Sensitivity analysis of the associations between serum folate and lipid abnormality subtypes after excluding participants using lipid-lowering therapy (*n*=3,181)

Characteristics	Model 1		Model 2		Model 3	
	OR (95% CI)	*p*	OR (95% CI)	*p*	OR (95% CI)	*p*
Hypercholesterolemia						
Serum folate per SD increment	1.00(0.88-1.14)	0.949	0.95(0.82-1.09)	0.425	0.94(0.82-1.08)	0.373
Serum folate quartile						
Q1 (*n*=794)	1.00(Reference)		1.00(Reference)		1.00(Reference)	
Q2 (*n*=795)	0.81(0.56-1.17)	0.256	0.74(0.51-1.08)	0.115	0.72(0.50-1.06)	0.095
Q3 (*n*=795)	0.98(0.69-1.40)	0.922	0.85(0.59-1.23)	0.380	0.84(0.58-1.21)	0.342
Q4 (*n*=797)	0.85(0.59-1.23)	0.390	0.70(0.47-1.03)	0.068	0.68(0.46-1.00)	0.051
p for trend		0.604		0.148		0.120
Hypertriglyceridemia						
Serum folate per SD increment	0.78(0.70-0.87)	<0.001***	0.78(0.69-0.87)	<0.001***	0.79(0.70-0.89)	<0.001***
Serum folate quartile						
Q1 (*n*=794)	1.00(Reference)		1.00(Reference)		1.00(Reference)	
Q2 (*n*=795)	0.90(0.70-1.15)	0.401	0.88(0.68-1.13)	0.304	0.87(0.67-1.13)	0.296
Q3 (*n*=795)	0.75(0.58-0.97)	0.030*	0.73(0.56-0.96)	0.022*	0.75(0.57-0.99)	0.043*
Q4 (*n*=797)	0.56(0.43-0.74)	<0.001***	0.54(0.41-0.73)	<0.001***	0.56(0.42-0.76)	<0.001***
p for trend		<0.001***		<0.001***		<0.001***
High LDL-C						
Serum folate per SD increment	1.07(0.91-1.25)	0.441	1.02(0.86-1.21)	0.845	1.03(0.87-1.23)	0.728
Serum folate quartile						
Q1 (*n*=794)	1.00(Reference)		1.00(Reference)		1.00(Reference)	
Q2 (*n*=795)	0.97(0.59-1.59)	0.895	0.90(0.54-1.49)	0.676	0.87(0.53-1.45)	0.600
Q3 (*n*=795)	1.16(0.72-1.87)	0.548	1.02(0.63-1.68)	0.922	1.02(0.62-1.68)	0.933
Q4 (*n*=797)	1.00(0.61-1.63)	0.988	0.85(0.50-1.42)	0.525	0.86(0.51-1.46)	0.575
p for trend		0.936		0.584		0.677
Low HDL-C						
Serum folate per SD increment	0.59(0.46-0.76)	<0.001***	0.62(0.48-0.80)	<0.001***	0.61(0.47-0.80)	<0.001***
Serum folate quartile						
Q1 (*n*=794)	1.00(Reference)		1.00(Reference)		1.00(Reference)	
Q2 (*n*=795)	0.57(0.37-0.88)	0.011*	0.57(0.37-0.89)	0.014*	0.56(0.36-0.88)	0.011*
Q3 (*n*=795)	0.43(0.27-0.69)	<0.001***	0.45(0.28-0.73)	0.001**	0.45(0.28-0.74)	0.001**
Q4 (*n*=797)	0.31(0.18-0.53)	<0.001***	0.33(0.19-0.58)	<0.001***	0.33(0.19-0.58)	<0.001***
p for trend		<0.001***		<0.001***		<0.001***

**p*<0.05; ***p*<0.01; ****p*<0.001.

Abbreviations: SD, standard deviation; LDL-C, low density lipoprotein cholesterol; HDL-C, high density lipoprotein cholesterol; OR, odds ratio; CI, confidence interval; Q1, first quartile; Q2, second quartile; Q3, third quartile; Q4, fourth quartile.

Model 1: unadjusted analysis (no covariates included); Model 2: adjusted for age, sex; Model 3 was adjusted for age, sex, education level, body mass index, serum vitamin B12, hypertension, diabetes, smoking, alcohol drinking, and regular exercise.

Table S3. Sensitivity analysis of the associations between serum folate and lipid abnormality subtypes without adjustment for vitamin B12 (*n* = 3,254)

Characteristics	OR (95% CI)	*p*
Hypercholesterolemia		
Serum folate per SD increment	0.94(0.82-1.08)	0.397
Serum folate quartile		
Q1 (*n*=810)	1.00(Reference)	
Q2 (*n*=815)	0.72(0.50-1.05)	0.085
Q3 (*n*=814)	0.86(0.60-1.24)	0.419
Q4 (*n*=815)	0.70(0.48-1.02)	0.063
p for trend		0.153
Hypertriglyceridemia		
Serum folate per SD increment	0.80(0.71-0.90)	<0.001***
Serum folate quartile		
Q1 (*n*=810)	1.00(Reference)	
Q2 (*n*=815)	0.84(0.65-1.08)	0.171
Q3 (*n*=814)	0.71(0.54-0.93)	0.013*
Q4 (*n*=815)	0.58(0.44-0.78)	<0.001***
p for trend		<0.001***
High LDL-C		
Serum folate per SD increment	1.03(0.87-1.22)	0.746
Serum folate quartile		
Q1 (*n*=810)	1.00(Reference)	
Q2 (*n*=815)	0.88(0.53-1.44)	0.605
Q3 (*n*=814)	1.02(0.63-1.66)	0.934
Q4 (*n*=815)	0.87(0.52-1.45)	0.588
p for trend		0.692
Low HDL-C		
Serum folate per SD increment	0.63(0.49-0.81)	<0.001***
Serum folate quartile		
Q1 (*n*=810)	1.00(Reference)	
Q2 (*n*=815)	0.52(0.33-0.80)	0.003**
Q3 (*n*=814)	0.43(0.27-0.70)	<0.001***
Q4 (*n*=815)	0.37(0.22-0.63)	<0.001***
p for trend		<0.001***

*$p<0.05$; **$p<0.01$; ***$p<0.001$.

Abbreviations: SD, standard deviation; LDL-C, low density lipoprotein cholesterol; HDL-C, high density lipoprotein cholesterol; OR, odds ratio; CI, confidence interval; Q1, first quartile; Q2, second quartile; Q3, third quartile; Q4, fourth quartile.

Adjusted for age, sex, education level, body mass index, hypertension, diabetes, smoking, alcohol drinking, regular exercise and lipid-lowering therapy.

Table S4. Sensitivity analysis of the associations between serum folate and lipid abnormality subtypes without adjustment for lipid-lowering therapy (*n* = 3,254)

Characteristics	OR (95% CI)	*p*
Hypercholesterolemia		
Serum folate per SD increment	0.94(0.82-1.08)	0.372
Serum folate quartile		
Q1 (*n*=810)	1.00(Reference)	
Q2 (*n*=815)	0.72(0.49-1.04)	0.080
Q3 (*n*=814)	0.86(0.60-1.24)	0.421
Q4 (*n*=815)	0.69(0.47-1.02)	0.060
p for trend		0.150
Hypertriglyceridemia		
Serum folate per SD increment	0.82(0.73-0.92)	<0.001***
Serum folate quartile		
Q1 (*n*=810)	1.00(Reference)	
Q2 (*n*=815)	0.86(0.67-1.11)	0.257
Q3 (*n*=814)	0.75(0.57-0.97)	0.031*
Q4 (*n*=815)	0.62(0.47-0.83)	<0.001***
p for trend		<0.001***
High LDL-C		
Serum folate per SD increment	1.02(0.86-1.22)	0.781
Serum folate quartile		
Q1 (*n*=810)	1.00(Reference)	
Q2 (*n*=815)	0.87(0.53-1.43)	0.589
Q3 (*n*=814)	1.01(0.62-1.65)	0.957
Q4 (*n*=815)	0.86(0.51-1.44)	0.561
p for trend		0.666
Low HDL-C		
Serum folate per SD increment	0.63(0.49-0.82)	<0.001***
Serum folate quartile		
Q1 (*n*=810)	1.00(Reference)	
Q2 (*n*=815)	0.53(0.34-0.82)	0.004**
Q3 (*n*=814)	0.44(0.27-0.71)	<0.001***
Q4 (*n*=815)	0.38(0.22-0.64)	<0.001***
p for trend		<0.001***

*$p<0.05$; **$p<0.01$; ***$p<0.001$.

Abbreviations: SD, standard deviation; LDL-C, low density lipoprotein cholesterol; HDL-C, high density lipoprotein cholesterol; OR, odds ratio; CI, confidence interval; Q1, first quartile; Q2, second quartile; Q3, third quartile; Q4, fourth quartile.

Adjusted for age, sex, education level, body mass index, serum vitamin B12, hypertension, diabetes, smoking, alcohol drinking, and regular exercise.

Table S5. Sensitivity analysis of the associations between serum folate and lipid abnormality subtypes using an extended adjustment model (*n* = 3,254)

Characteristics	OR (95% CI)	*p*
Hypercholesterolemia		
Serum folate per SD increment	0.95(0.83-1.09)	0.465
Serum folate quartile		
Q1 (*n*=810)	1.00(Reference)	
Q2 (*n*=815)	0.72(0.50-1.05)	0.086
Q3 (*n*=814)	0.86(0.60-1.24)	0.421
Q4 (*n*=815)	0.70(0.48-1.03)	0.072
p for trend		0.174
Hypertriglyceridemia		
Serum folate per SD increment	0.81(0.72-0.91)	<0.001***
Serum folate quartile		
Q1 (*n*=810)	1.00(Reference)	
Q2 (*n*=815)	0.86(0.67-1.12)	0.265
Q3 (*n*=814)	0.73(0.56-0.96)	0.024*
Q4 (*n*=815)	0.60(0.45-0.80)	<0.001***
p for trend		<0.001***
High LDL-C		
Serum folate per SD increment	1.06(0.89-1.27)	0.488
Serum folate quartile		
Q1 (*n*=810)	1.00(Reference)	
Q2 (*n*=815)	0.87(0.53-1.44)	0.589
Q3 (*n*=814)	1.02(0.63-1.67)	0.927
Q4 (*n*=815)	0.92(0.55-1.54)	0.743
p for trend		0.888
Low HDL-C		
Serum folate per SD increment	0.65(0.51-0.84)	<0.001***
Serum folate quartile		
Q1 (*n*=810)	1.00(Reference)	
Q2 (*n*=815)	0.55(0.35-0.86)	0.009**
Q3 (*n*=814)	0.43(0.26-0.69)	<0.001***
Q4 (*n*=815)	0.39(0.23-0.66)	<0.001***
p for trend		<0.001***

*$p<0.05$; **$p<0.01$; ***$p<0.001$.

Abbreviations: SD, standard deviation; LDL-C, low density lipoprotein cholesterol; HDL-C, high density lipoprotein cholesterol; OR, odds ratio; CI, confidence interval; Q1, first quartile; Q2, second quartile; Q3, third quartile; Q4, fourth quartile.

Adjusted for age, sex, education level, body mass index, serum vitamin B12, hypertension, diabetes, smoking, alcohol drinking, regular exercise, lipid-lowering therapy, vegetable intake, staple food intake, and self-rated economic status.

Table S6. Original and Firth-corrected logistic regression results for the associations of serum folate per 1-SD increment with high LDL-C and low HDL-C (*n* = 3,254)

Outcome	Original OR (95% CI)	Original *p* value	Firth-corrected OR (95% CI)	Firth-corrected *p* value
High LDL-C	1.03(0.86-1.22)	0.777	1.03(0.86-1.22)	0.727
Low HDL-C	0.63(0.49-0.81)	<0.001***	0.64(0.49-0.82)	<0.001***

***p<0.001.

Abbreviations: SD, standard deviation; OR, odds ratio; CI, confidence interval; LDL-C, low density lipoprotein cholesterol; HDL-C, high density lipoprotein cholesterol.

Adjusted for age, sex, education level, body mass index, serum vitamin B12, hypertension, diabetes, smoking, alcohol drinking, regular exercise and lipid-lowering therapy.

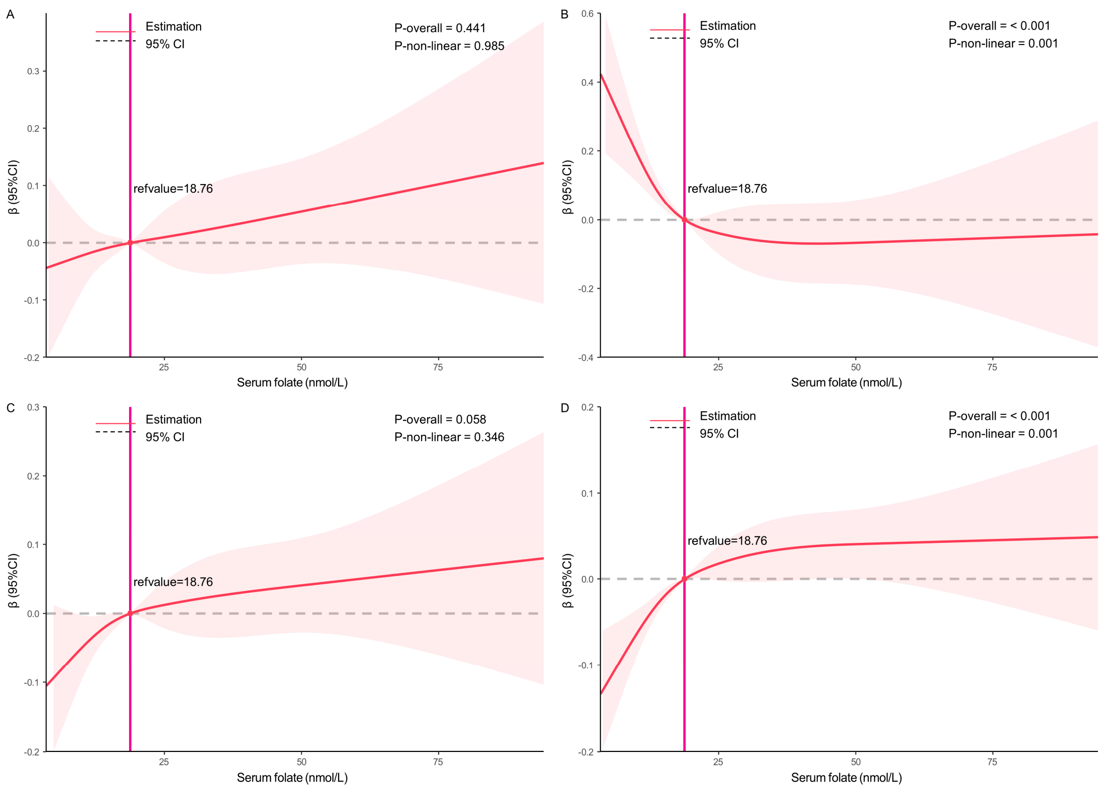

Figure S1. Dose-response relationships between serum folate and continuous lipid parameters. Restricted cubic spline analyses based on linear regression models with four knots were performed to assess the associations between serum folate and continuous lipid parameters, including total cholesterol (TC), triglycerides (TG), low-density lipoprotein cholesterol (LDL-C), and high-density lipoprotein cholesterol (HDL-C). Models were adjusted for age, sex, education level, body mass index (BMI), smoking, drinking, hypertension, diabetes, serum vitamin B12, regular exercise, and lipid-lowering therapy. Serum folate levels (nmol/L) are shown on the x-axis, and adjusted β coefficients with 95% confidence intervals (CIs) are shown on the y-axis, using the median serum folate concentration as the reference value. The solid red lines represent the fitted spline curves, and the shaded areas indicate the 95% CIs. The horizontal dashed line indicates a β coefficient of 0, representing no difference in lipid concentration compared with the reference value. Vertical solid lines indicate the 50th percentile (median; reference value) of the serum folate distribution. (A) Association between serum folate and TC. (B) Association between serum folate and TG. (C) Association between serum folate and LDL-C. (D) Association between serum folate and HDL-C. Abbreviations: CI, confidence interval.

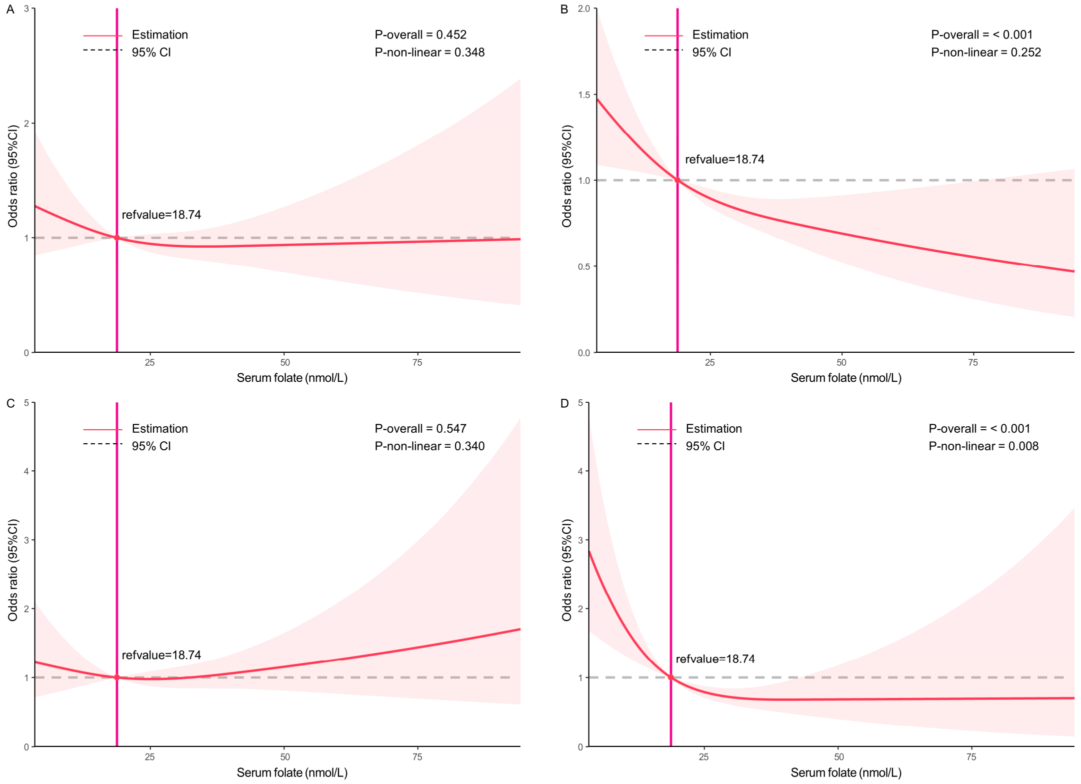

Figure S2. Dose-response relationships between serum folate and four lipid abnormality subtypes after excluding lipid-lowering therapy users. Restricted cubic spline regression models with four knots were used to examine the dose-response relationships between serum folate and lipid abnormality subtypes, including hypercholesterolemia, hypertriglyceridemia, high low-density lipoprotein cholesterol (LDL-C), and low high-density lipoprotein cholesterol (HDL-C), with adjustment for age, sex, education level, body mass index (BMI), smoking, drinking, hypertension, diabetes, serum vitamin B12, and regular exercise. Odds ratios (ORs) and 95% confidence intervals (CIs) were estimated from logistic regression models. Serum folate levels (nmol/L) are shown on the x-axis, and ORs for each lipid outcome are shown on the y-axis. The solid red lines represent the fitted spline curves, and the shaded areas indicate the 95% confidence intervals. The horizontal dashed line indicates an OR of 1 (no association). Vertical solid lines indicate the 50th percentile (median; reference value) of the serum folate distribution. (A) Association between serum folate and hypercholesterolemia. (B) Association between serum folate and hypertriglyceridemia. (C) Association between serum folate and high LDL-C. (D) Association between serum folate and low HDL-C. Abbreviations: OR, odds ratio; CI, confidence interval.
